# *Anthyllis apennina* (Fabaceae), a new species from central Apennine (Italy)

**DOI:** 10.3897/phytokeys.176.62774

**Published:** 2021-04-19

**Authors:** Fabio Conti, Fabrizio Bartolucci

**Affiliations:** 1 Scuola di Bioscienze e Medicina Veterinaria, Università di Camerino – Centro Ricerche Floristiche dell’Appennino, Parco Nazionale del Gran Sasso e Monti della Laga, San Colombo, 67021 Barisciano (L’Aquila), Italy Università di Camerino Camerino Italy

**Keywords:** Abruzzo, *Anthyllis vulneraria* species complex, Italian endemics, Lazio, Loteae, Papilionoideae, taxonomy

## Abstract

A new species of *Anthyllis* endemic to central Apennine growing in dry pastures on limestone in the montane belt, within Abruzzo and Lazio administrative regions (central Italy), is here described and illustrated and the IUCN assessment is proposed. This new species belongs to the morphologically very variable *Anthyllis vulneraria* species complex and it is close to *A. pulchella* (south-eastern Europe and Caucasus), but it can be clearly distinguished by its smaller flowers, mainly light yellow-coloured, bracts longer than flowers, calyx pink-coloured (usually only at apex) and size of cauline leaves and leaflets. Furthermore, the name *A. pulchella* is here lectotypified, by a second-step typification, on a specimen preserved at PAD.

## Introduction

The genus *Anthyllis* L. belongs to subfamily Papilionoideae, tribe Loteae (Polhill 1981; Allan and Porter 2000; Degtjareva et al. 2012). The *A. vulneraria* species complex, distributed in Europe, Mediterranean Basin, Turkey, Caucasus, Iran, Eritrea and Ethiopia, is morphologically very variable and several infraspecific taxa, many of them frequently regarded as independent species, have been recognised (e.g. Cullen 1968, 1976; Akeroyd 1988; Roskov et al. 2006; Rola 2012; POWO 2019). Roskov et al. (2006) reported from Europe and Mediterranean countries 29 infraspecific taxa. Further taxa are quoted by Lampinen (1990), Benedí (2000), Greuter and Raus (2005), Puidet et al. (2005), Tison and Foucault (2014) and Conti et al. (2016). With regards to Italy, the *A. vulneraria* complex is represented by 15 subspecies, of which two are endemic and one is alien (Bartolucci et al. 2018; Galasso et al. 2018). On the other hand, Pignatti et al. (2017, 2019) recognise the taxa belonging to this group as species and quoted for Italy 13 species (two alien), one subspecies and five hybrids. In Italy, according to our field observations, many taxa are clearly sympatric, growing in the same localities and habitat. These taxa are usually clearly distinct and, probably, to be considered as separate and independent species. The lack of nomenclatural, taxonomic and molecular studies regarding this species complex has led to unclear descriptions of taxa and to unworkable analytical keys. We believe that some taxa should be recognised at species rank as proposed by Pignatti et al. (2019), but further studies are still needed. During the fieldwork concerning the “Flora of Abruzzo” project, one of us (FC) came across a population of *Anthyllis* on Mt. Ocre showing peculiar morphological features. Later, we found other populations with plants characterized by the same features on Mt. Boragine and Selva Rotonda (Lazio, Reatini Mountains) during the annual field trip of the working group for Floristics, Systematics and Evolution of the Italian Botanical Society held in 2016 (Bartolucci et al. 2019). Additional populations were discovered on the southern slopes of Pizzo Camarda and between S. Stefano di Sessanio and S. Colombo (Gran Sasso massif), Vallone di Sevice and Piani di Pezza (Mt. Velino), Mt. Sirente, Mt. Calvo and in some localities of the National Park of Abruzzo, Lazio and Molise (Mt. Tricella, Colle Biferno, La Brecciosa). Plants of these populations are morphologically different from other known taxa within the *A. vulneraria* species complex, showing a peculiar combination of characters as very small flowers, mainly light yellow-coloured, and bracts longer than flowers. They are confused in the past with the close *A. pulchella* (Vis.) Beck (≡ A. vulneraria L. subsp. pulchella (Vis.) Bornm.), a species distributed in south-eastern Europe, Crimea and Caucasus (Cullen 1976; Akeroyd 1986; Dimopoulos et al. 2013; Barina et al. 2018; Bartolucci et al. 2018; Pignatti et al. 2019). Two other similar taxa, but clearly distinguished, are the endemic to central Apennine A. vulneraria subsp. nana (Ten.) Tammaro (Conti et al. 2016) and the western European A. vulneraria subsp. vulnerarioides (All.) Arcang. (Benedí 2000; Tison and Foucault 2014; Conti et al. 2016). Field investigations and an extensive morphological study on herbarium material, providing evidence about the species differentiation between *A. pulchella* and the Apennine populations, have been carried out. The results allowed us to describe the Apennine populations as a new species with the name *A. apennina*.

## Material and methods

**Figure 1.**
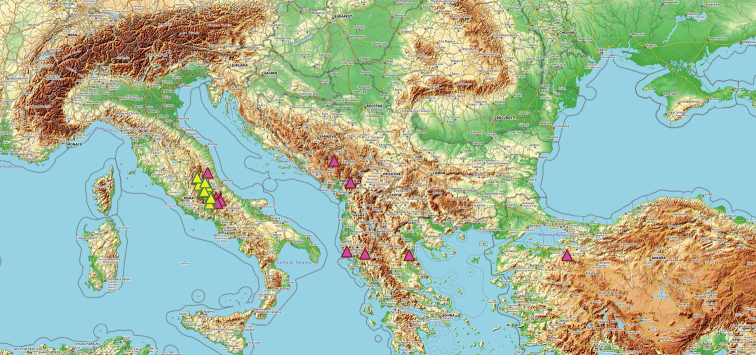
Distribution map of *Anthyllis apennina* and *A. pulchella* according to the analyzed material.

This study is based mainly on field surveys, on an extensive analysis of relevant literature and on examination of herbarium specimens (including nomenclatural types) preserved at APP, B, BM, GAP, P, PAD, TO (codes following Thiers 2020). A total of 125 specimens from these herbaria were studied. Ad hoc sampling campaigns in the central Apennines to better understand the distribution of the new species were carried out. The herbarium specimens of A. vulneraria subsp. vulnerarioides and A. vulneraria subsp. nana were studied for a preliminary morphological circumscription of the species, and not used for morphological analyses. A more detailed morphological comparison involved *A. apennina* and the closest species *A. pulchella*. Morphological observations and measurements of qualitative and quantitative characters, considered as diagnostic in *Anthyllis* (e.g., Cullen 1968, 1976; Akeroyd 1988; Rola 2012), were analysed on dried specimens. The analyses were performed on 58 variables, including 40 quantitative continuous characters, 6 quantitative discrete characters, 2 ratios and 10 qualitative characters (Table 1). The morphometric analyses were carried out on 37 selected specimens including *A. apennina* (21 specimens) and *A. pulchella* (16 specimens). Each flower was soaked in water for a few seconds before taking the measurements, using the maximum parameters. Calyx length include hairs protruding upper teeth. The calyx was sectioned longitudinally and then the width of its entire development was measured. Flower colour is based on living specimens because it was not possible to evaluate this character reliably from herbarium specimens. All morphological characters were observed with a Leica MZ16 stereomicroscope. The herbarium specimens used for the morphometric analyses are shown in Fig. 1 (produced using QGIS 3.16.4).

**Table 1.** Morphological characters employed in the morphometric analyses.

	Abbreviations	Characters
1	HB	Habit (erect=1; straggling=2)
2	SH	Stem height (mm)
3	SHAB	Stem hairness below (appressed=1; patent=2)
4	SHAA	Stem hairness above (appressed=1; patent=2)
5	SLL	Shape of lower leaves (equal leaflets=1; subequal=2; unequal =3)
6	LAFS	Leaves arrangement along the flowering stem (equal=1; mainly at base=2)
7	NSL	N. of stem leaves
8	HALB	Hairness of the upper leaf blade (glabrous=1; hairy=2)
9	HAP	Hairness of petiole (appressed=1; patent=2)
10	BLL	Basal leaf length (mm)
11	NBLL	N. leaflets in basal leaf
12	TBLLL	Terminal leaflet length in basal leaf (mm)
13	TBLLW	Terminal leaflet width in basal leaf (mm)
14	LBLLL	Lateral leaflet length in basal leaf (mm)
15	LBLLW	Lateral leaflet width in basal leaf (mm)
16	LCLL	Lower cauline leaf length (mm)
17	NLCLL	N. of leaflets in lower cauline leaves
18	TLCLL	Terminal leaflet length in lower cauline leaf (mm)
19	TLCLW	Terminal leaflet width in lower cauline leaf (mm)
20	LLCLL	Lateral leaflet length in lower cauline leaf (mm)
21	LLCLW	Lateral leaflet width in lower cauline leaf (mm)
22	UCLL	Upper cauline leaf length (mm)
23	NUCLL	N. of leaflets in upper cauline leaf
24	TUCLLL	Terminal leaflet length in upper cauline leaf (mm)
25	TUCLLW	Terminal leaflet width in upper cauline leaf (mm)
26	LUCLLL	Lateral leaflet length in upper cauline leaf (mm)
27	LUCLLW	Lateral leaflet width in upper cauline leaf (mm)
28	NINFL	N. of inflorescences for each stem
29	BL	Bract length (mm)
30	BLL	Bract lobe length (mm)
31	NB	N. of bract lobes
32	BA	Bract apex (acute-apiculate=1; obtuse=2)
33	CI	Calyx indumentum (appressed=1; semipatent-patent=2)
34	CC	Calyx colour (apex concolour=1; pink-purple=2; purple=3)
35	CL	Calix length (hairs included) (mm)
36	CW	Calyx width (mm)
37	BL/CL	Ratio bract/calyx length
38	UCTL	Upper calyx teeth length (mm)
39	UCTW	Upper calyx teeth width (mm)
40	LCTL	Lateral calyx teeth length (mm)
41	LCTW	Lateral calyx teeth width (mm)
42	LOCTL	Lower calyx teeth length (mm)
43	LOCTW	Lower calyx teeth width (mm)
44	UCTHAL	Hairs length on upper calyx teeth (mm)
45	LCTHAL	Hairs length on lower calyx teeth (mm)
46	MCHAL	Hairs length in the middle of calyx (mm)
47	SL	Standard length (mm)
48	SW	Standard width (mm)
49	SCL	Standard claw length (mm)
50	BL/SL	Ratio bract/standard length
51	WL	Wing length (mm)
52	WW	Wing width (mm)
53	WCL	Wing claw length (mm)
54	KL	Keel length (mm)
55	KW	Keel width (mm)
56	KCL	Keel claw length (mm)
57	STL	Staminal tube length (without free filaments and anthers) (mm)
58	SPL	Stipe of pod length (mm)

For each quantitative character, Shapiro-Wilks normality test was first used to determine their distribution, then an independent sample T-test, after logarithmic transformation, was carried out with SPSS version 25 (IBM 2017). Principal coordinate analysis (PCoA) and cluster analysis (UPGMA) were performed in PAST package version 4.03 (Hammer et al. 2001; Hammer 2020). Furthermore, the variability of the analysed morphological characters was described by standard statistical parameters (mean, standard deviation, minimum, maximum, 10^th^ and 90^th^ percentiles). Boxplots were built by means of SPSS version 25 (IBM 2017).

For the lectotype selection of the name *A. pulchella*, the protologue has been compared with original material and the most complete and informative specimen was selected (Art. 9.4, Turland et al. 2018).

The conservation assessment according to the IUCN Criteria is proposed and briefly discussed (IUCN 2019).

## Taxonomy

### Anthyllis apennina

Taxon classificationPlantaeFabalesFabaceae

F.Conti & Bartolucci sp. nov.

CC54F224-1C21-583A-A766-7B4FFD7D2DFF

urn:lsid:ipni.org:names:77216597-1

Figs 2 , 3

#### Diagnosis.

*Anthyllis apennina* differs from *A. pulchella* by the bigger cauline leaves and leaflets, leaves evenly distributed along the flowering stem vs. concentrated in lower half, higher number of inflorescences 2–5(–10) vs. 1–2, smaller flowers with standard (7.9–)8.5–9.9(–10.3) vs. (10–)10.4–13.6(–13.8) mm long, longer bracts (12–)14–23(–26) vs. (5.8–)6.5–11(–12) mm long, longer than flowers, with longer lobes (10–)11–21.5(–24) vs. (3.2–)3.9–7.5(–9) mm long, narrowly triangular and acute to apiculate vs. more or less parallel-sided and obtuse and by the colour of flowers which are mainly light yellow or flushed with pink vs. purplish-pink or cream flushed with pink and the calyx which is mainly pink only in the upper part.

#### Type.

Italy. Abruzzo, M. Tricella (Ortona dei Marsi), pascoli aridi, 1300 m a.s.l., 29/05/2017, *F. Bartolucci*, *F. Conti & L. Di Martino s.n.* (holotype APP No. 59652; isotypes APP Nos. 59645, 59648, 59650, 59651, 59653, 59666) (Fig. 3).

**Figure 2.**
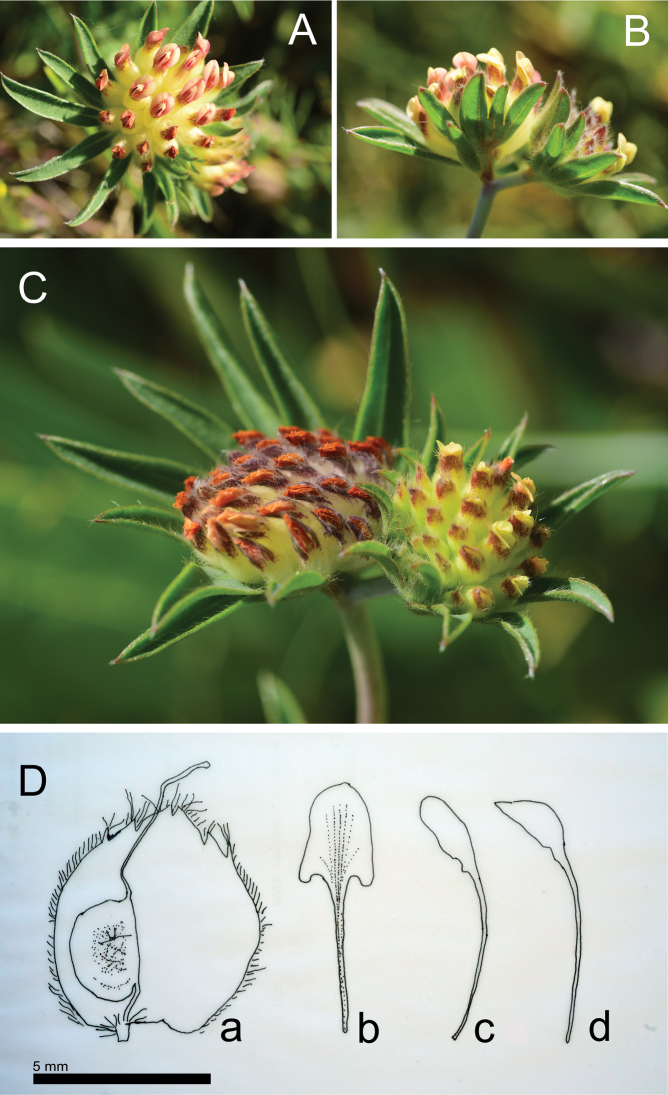
*Anthyllis apennina* F.Conti & Bartolucci, sp. nov. **A** inflorescence (Mt. Tricella, photo F. Conti) **B** bracts of inflorescences (Mt. Tricella, photo F. Conti) **C** geminate inflorescences (Prati del Sirente, photo F. Conti) **D** drawing from herbarium specimen APP No. 59887 collected on Colle Biferno **a** calyx and pod with stylus and stigma **b** standard **c** wing **d** keel.

**Figure 3.**
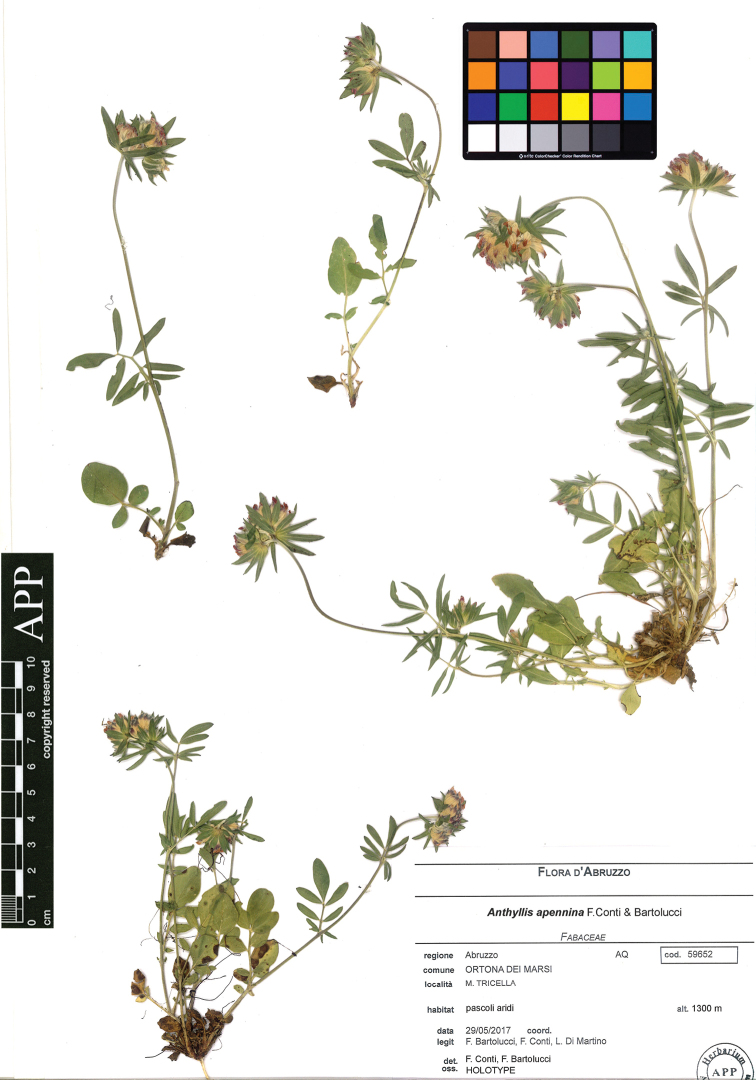
Holotype of *Anthyllis apennina* F.Conti & Bartolucci (APP No. 59652, reproduced with permission of the Herbarium, Centro Ricerche Floristiche dell’Appennino, Italy).

#### Description.

Annual, erect branched to straggling. Stem height (110–)160–330(–450) mm. Stem appressed sericeous over its whole length. Leaves alternate, pinnate, densely appressed hairy on the lower side, glabrous on the upper side (basal and lower cauline leaves) or sparsely hairy with long hairs (middle and upper cauline leaves), the basal ones usually withered at anthesis, (16–)17.6–35(–55) mm, reduced to a terminal leaflet or imparipennate with 2–5 leaflets with a much larger terminal leaflet elliptic, ovate or obovate, obtuse, (7–)8,9–19.1(–24) × (4–)5–13.1(14) mm, lateral leaflets 0–5.7(–9) × 0–2.9(–4.9) mm. Stem with 3–4(–6) leaves at subequal distances along the flowering stem. Lower cauline leaf (32–)42.8–69.4(–74) mm, imparipennate with 3–7 leaflets with a much larger terminal leaflet ovate or obovate, obtuse to acute, (15–)19.6–33.8(–40) × (8–)9.6–17.4(–22) mm, lateral leaflets (4–)4.6–15.6(–21) × (1.2–)2.1–5.4(–6) mm. Upper cauline leaves (24–)26–40(–44) mm, imparipennate with 7–13 leaflets subequal (equifoliolate), terminal leaflet narrowly elliptic to narrowly obovate, acute to apiculate, (13–)15–27(–29) × (2.5–)2.8–6(–8) mm, lateral leaflets (12.5–)14–25(–27) × (2–)2.2–4(–7) mm. Every stem with 2–5(–10) heads, many flowered subtended by 2 palmatisect bracts borne close beneath the flowers. Flowers light yellow or flushed with pink. Bracts (12–)14–23(–26) mm, lobes of bracts 3–7, (10–)11–21.5(24) mm, narrowly triangular, tapering, appressed hairy, acute to apiculate. Calyx (8.3–)8.5–10.4(–10.9) × (5.9–)6.2–7.8(–8.3) mm, whitish to yellowish, pink to pink-purple at apex, in dry specimens weakly pink, hairy subpatent, upper teeth triangular (0.3–)0.4–0.6(–0.9) mm at base, large (0.3–)0.4–0.9(–1) mm. Lateral teeth triangular (0.3–)0.4–0.7 mm, at base large (0.3–)0.4–0.7 mm. Lower tooth narrowly triangular to linear (0.5–)0.6–0.9(–1) × 0.3–0.6 mm. Standard (7.9–)8.5–9.9(–10.3) × (2.3–)2.6–3.3(–3.8) mm, standard claw (4.6–)5.4–6.1(–6.4) mm. Wing (8.1–)8.8–10.1(–10.5) × (1–)1.2–1.6(–1.7) mm, wing claw (5.3–)5.8–6.8(–7) mm long. Keel (8–)9–10.5(–10.8) × 1.1–1.4(–1.6) mm, keel claw 5.6–6.1(–7.4) mm long. Ratio bract length/standard length (1.5–)1.6–2.3(–2.5) and ratio bract length/calyx length (1.4–)1.6–2.3(–3.0). Staminal tube length (without free filaments and anthers) (6.6–)7.3–8.7(–8.8) mm. Stylus (3.8–)3.9–4.3(–4.4) mm. Stigma 1.9–2(-2.1) mm. Stipe of pod (0.9–)1–1.5(–1.8) mm. Legume 1-seeded 3.5–4 × 2.8–3 mm, seeds ca. 2 mm.

#### Etymology.

*Anthyllis apennina* is named after the Apennine to which the species is endemic.

#### Habitat.

Pastures in montane belt from 1200 to 1800 m a.s.l.

#### Phenology.

Flowering from the second half of April to the end of July, fruiting in June-July.

#### Distribution.

Central Apennine in Lazio and Abruzzo (Fig. 1).

#### Conservation status.

Some of the populations of *A. apennina* occurred in a NATURA 2000 network within the Sites of Community Interest “IT7110208 Monte Calvo e Colle di Macchialunga”, “IT7110206 Monte Sirente e Monte Velino”, “IT710202 Gran Sasso” and within the Abruzzo, Lazio and Molise National Park, Gran Sasso and Laga Mountains National Park and Sirente-Velino Regional Park. The extent of occurrence (EOO) is 1788 km^2^ calculated with the minimum convex hull polygon in QGIS and area of occupancy (AOO) is 52 km^2^ calculated with a 2×2 km cell fixed grid. No pressures or threats are evidenced. According to IUCN Criteria (IUCN 2019), we propose to include *A. apennina* in the following category: Least Concern (LC).

#### Morphometric analysis.

**Figure 4.**
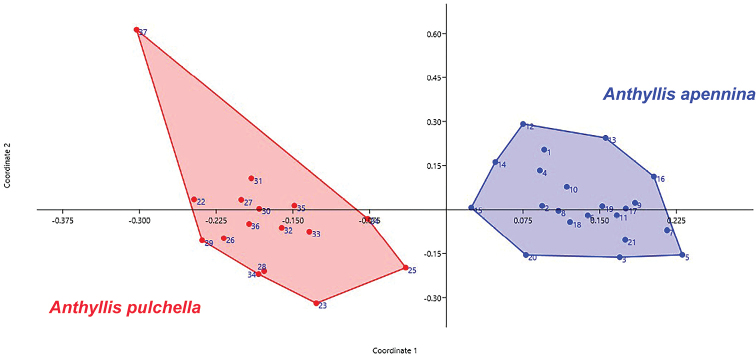
Scatter plot of first two principal coordinate axes based on 31 morphological characters and 37 specimens.

**Figure 5.**
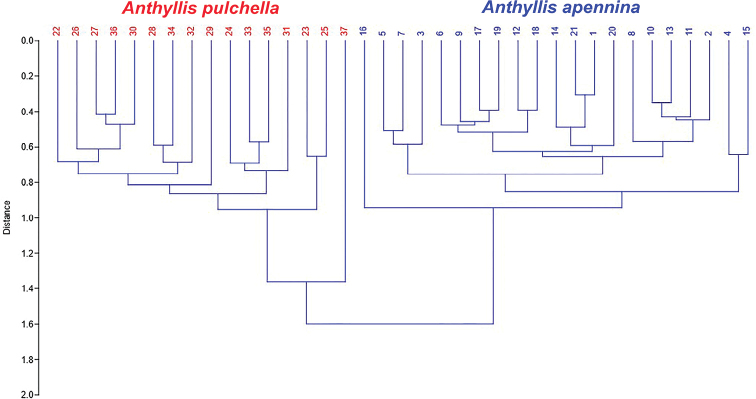
UPGMA dendrogram, showing the phenetic relationships among the 37 studied specimens of *Anthyllis apennina* and *A. pulchella*.

**Figure 6.**
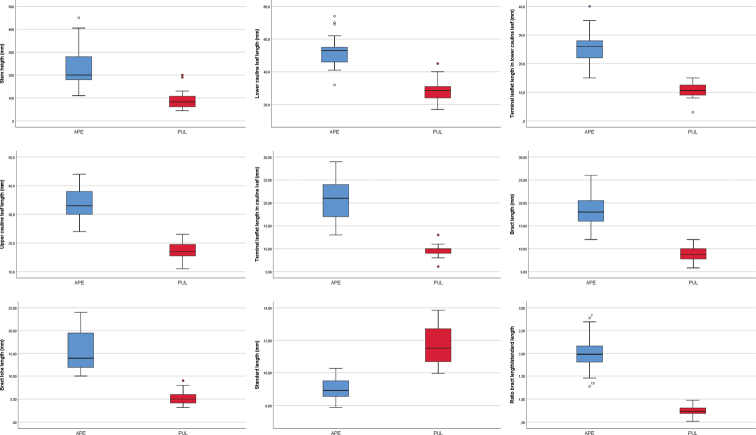
Boxplots expressing morphological variation between *Anthyllis apennina* (APE) and *A. pulchella* (PUL): stem height (mm), lower cauline leaf length (mm), terminal leaflet length in lower cauline leaf (mm), upper cauline leaf length (mm), terminal leaflet length in upper cauline leaf (mm), bract length (mm), bract lobe lenght (mm), standard length (mm), ratio bract length/standard length. Outlined central box depicts middle 50% of data, extending from 25^th^ and 75^th^ percentiles, and horizontal bar is the median. Ends of vertical lines (or “whiskers”) indicate minimum and maximum data values, unless outliers are present, in which case whiskers extend to a maximum of 1.5 times inter-quartile range. Circles indicate outliers.

The principal coordinate analysis (PCoA, Fig. 4) shows on the first two axes (explained variance: 57.1% and 11.1%) a clear separation between *A. apennina* and *A. pulchella*. Concerning the intraspecific variation within *A. pulchella*, one separated specimen (“37” from Turkey; see Fig. 1 and specimens examined) can be recognized in the PCoA diagram. Cluster analysis (UPGMA, Fig. 5), shows two well-delimited clusters supporting a clear separation between the studied species. Also in the UPGMA the specimen “37” from Turkey is separated. Most of the quantitative morphometric characters evaluated showed significant differences between the two *Anthyllis* species (Table 2). The most relevant morphological characters differentiating the two species are summarized in Table 3 and shown in Fig. 6.

**Table 2.** T score and P value of quantitative characters evaluated (significant P values at P < 0.05 in boldface).

Characters	T score	P value	Characters	T score	P value
SH	7.176	**0.000**	CW	1.271	0.216
BLL	3.554	**0.001**	UCTL	0.476	0.638
TBLLL	3.118	**0.004**	UCTW	-2.007	0.054
TBLLW	3.815	**0.001**	LCTL	-2.563	**0.016**
LBLLL	0.979	0.343	LCTW	0.132	0.896
LBLLW	0.526	0.609	LOCTL	-5.208	**0.000**
LCLL	7.431	**0.000**	LOCTW	-1.011	0.323
TLCLL	8.724	**0.000**	UCTHAL	0.839	0.411
TLCLW	6.716	**0.000**	LCTHAL	1.03	0.314
LLCLL	2.999	**0.006**	MCHAL	-1.802	0.085
LLCLW	1.747	0.091	SL	-8.533	**0.000**
UCLL	9.658	**0.000**	SW	-5.469	**0.000**
TUCLLL	11.663	**0.000**	SCL	-5.273	**0.000**
TUCLLW	3.721	**0.001**	WL	-6.15	**0.000**
LUCLLL	9.418	**0.000**	WW	-6.268	**0.000**
LUCLLW	5.909	**0.000**	WCL	-4.335	**0.000**
BL/CL	15.452	**0.000**	KL	-5.96	**0.000**
BL/SL	17.547	**0.000**	KW	-3.732	**0.001**
BL	10.573	**0.000**	KCL	-3.79	**0.001**
BLL	11.618	**0.000**	STL	-5.516	**0.000**
CL	-4.774	**0.000**	SPL	-2.447	**0.024**

#### Typification of the name *Anthyllis pulchella*.

Visiani (1872: 141) described A. vulneraria var. pulchella providing a short description, and reporting the following type localities: “in monte Orien, et Montenegro in monte Lovçen ad alt. ped. 6000: Legit rev. Huter”. Referring to the “type”, Cullen (1970) cited several syntypes of the gathering by Huter on Mt. Lovćen kept in BM, K, P, and W. The Cullen’s typification (1970) satisfies the Arts. 7.10 and 7.11 of the ICN (Turland et al. 2018), but he did not cite a single specimen as type. However, it is possible to consider the Cullen typification as a “first-step lectotypification”,that may be further narrowed to a single specimen by a “second-step lectotypification” according to Art. 9.17 of the ICN. We performed a survey for original material at PAD, where the De Visiani’s main collection is housed (Stafleu and Cowan 1986) and in the above mentioned herbaria. We were able to trace some duplicate specimens of the gathering on Mt. Lovćen in PAD (barcode HD08734), B (barcode B 10 111324) and BM (barcodes BM000751284 and BM000751285), which can be considered for the second-step typification. These herbarium specimens are complete, well conserved and agree with the protologue and with the current application of the name (Cullen 1968, 1970, 1976; Akeroyd 1986; Bartolucci et al. 2018). The herbarium specimen kept at PAD with barcode HD08734 is selected here as second-step lectotype.

Other names, some of which having priority at species rank, as *A. scardica* Wettst., *A. albana* Wettst., *A. biebersteiniana* Popl., and *A. daghestanica* Chinth. are regarded by some authors (e.g. Roskov et al. 2006) as synonyms of *A. pulchella*. These taxa need further study and/or typification.

Anthyllis vulneraria var. pulchella Vis., Fl. Dalmat. Supplementum: 141. 1872 ≡ *Anthyllis pulchella* (Vis.) Beck, Ann. K. K. Naturhist. Hofmus. 11: 65. 1896 ≡ Anthyllis vulneraria subsp. pulchella (Vis.) Bornm., Bot. Jahrb. Syst. 59: 483. 1925.

- Anthyllis vulneraria var. pusilla Vis., in schedis
- Anthyllis vulneraria var. pauciflora Asch. & Huter, in schedis

Type (second-step lectotype, here designated; first-step designated by Cullen 1970: 536): Montenegro, Lovcen, 6000, 5/06/1867, *Huter s.n.* (PAD barcode HD08734!; isolectotypes B barcode B101113247!, BM barcodes BM000751284! and BM000751285!).

**Figure 7.**
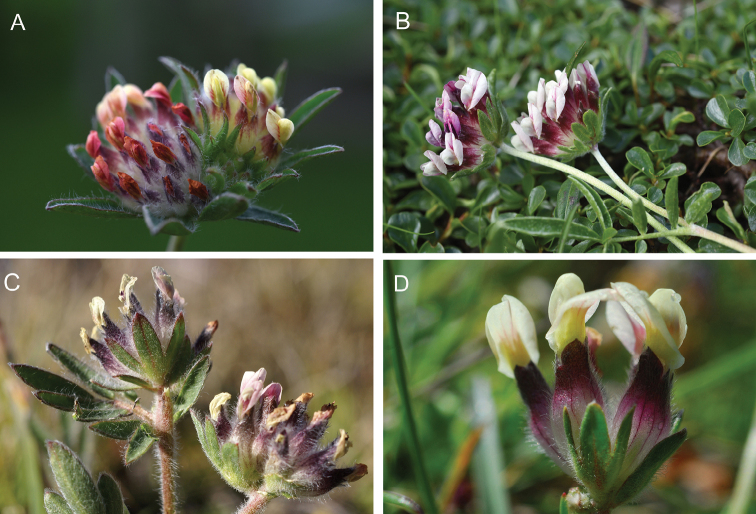
**A***Anthyllis apennina*, Mt. Tricella, Abruzzo, Italy (photo F. Conti) **B**Anthyllis vulneraria subsp. nana, Monte Focalone, Abruzzo, Italy (photo F. Conti) **C**Anthyllis vulneraria subsp. vulnerarioides, Mont Cenis, Francia (photo G. Pache) **D***Anthyllis pulchella*, Campo Felice, Abruzzo, Italy (photo F. Conti).

**Discussion.** According to Cullen (1976), in the *A. vulneraria* species complex, it is possible to recognise *Alpestris* and *Vulneraria* aggregates. The new species, according to some morphological characters, such as the upper cauline leaves equifoliolate, lobes of bracts tapering, acute at apex and lateral calyx teeth obscure, adpressed to the upper, belongs to the *Vulneraria* aggregate. The greatest diversity of the complex was found in western and southern Europe: Spain, France and Italy (Roskov et al. 2006). *Anthyllis apennina* can be clearly distinguished from species belonging to *A. vulneraria* complex by small flowers, mainly light yellow-coloured, and bracts longer than flowers. The most similar taxon is *A. pulchella*, a SE European species, occurring in central Apennine and rarely sympatric with *A. apennina* which differs due to its smaller cauline leaves with smaller terminal leaflet, lower number of inflorescences (1–2 vs. 2–10), less divided and smaller bract (5.8–12 mm vs. 12–26 mm), bract shorter than flower (vs. longer than flower), smaller standard, wing and keel. For *A. pulchella*, Akeroyd (1986) reports a corolla, even larger than what we measured, to be 12–14(17) mm long. Furthermore, the calyx in *A. apennina* is smaller and pink-purple only at the apex, while in *A. pulchella*, it is purple at the apex and the coloured part is longer (see Table 3). Other similar taxa are: A. vulneraria subsp. nana, endemic to central Apennine, that grows in stony pastures, usually above treeline in the alpine belt, which differs from *A. apennina* due to its larger corollas 13–17 mm [vs. (8–)9–10.5(–10.8) mm] usually whitish or pink (vs. light yellow or flushed with pink), and bracts smaller than flowers (vs. longer than flowers); A. vulneraria subsp. vulnerarioides, a western European species, described from Moncenisio and not occurring in central Apennine (Conti et al. 2016) characterized by stems with patent hairs (vs. appressed sericeous over its whole length), basal leaves hairy on the upper surface (vs. glabrous on the upper side), with 5–11 leaflets and the terminal one slightly larger than the lateral ones (Tison and Foucault 2014; Conti et al. 2016; Fig. 7). Further studies to clarify the taxonomic position of the several taxa currently included within *A. vulneraria* species complex are needed. The new endemic species enriches the already considerable floristic heritage of the central Apennine. Abruzzo Region, which includes the main peaks of the central Apennines, is the fourth Italian Region, after Sicily, Sardinia and Calabria by number of endemics, but with this new taxon become the third region together with Calabria (Bartolucci et al. in press). This number has increased in recent years thanks to the activity of the Floristic Research Centre of the Apennines (Conti 2007, 2010; Conti and Peruzzi 2006; Peruzzi et al. 2007; Conti and Uzunov 2011; Gallo and Conti 2015; Conti and Bartolucci 2017; Conti et al. 2018, 2019, 2020).

**Table 3.** Main diacritic features of *Anthyllis apennina* and *A. pulchella*. Quantitative continuous characters are expressed in mm and are reported as mean ± standard deviation and 10–90 percentiles (extreme values in brackets). For quantitative discrete cardinal characters, 10–90 percentiles are given (extreme values in brackets).

Character	*Anthyllis apennina*	*Anthyllis pulchella*
Stem height	(110–)160–330(–450)	(45–)60–160(–200)
	237.4±88.2	95.6±44.6
Leaves arrangement along the flowering stem	evenly distributed	concentrated in lower half
Lower cauline leaf length	53.2±10.8	28.5±7.8
	(32–)42.8–69.4(–74)	(17–)18–39(–45)
Terminal leaflet length in lower cauline leaf	26.3±6.2	10.4±2.9
	(15–)19.6–33.8(–40)	(3–)8–13.5(–15)
Terminal leaflet width in lower cauline leaf	13.4±3.6	5.5±1.8
	(8–)9.7–17.4(–22)	(1–)3.7–7.2(–8)
Upper cauline leaf length	33.6±5.6	17.3±3.8
	(24–)26–40(–44)	(11–)12–22.2(–23)
Terminal leaflet length in upper cauline leaf	21.1±4.5	9.6±1.6
	(13–)15–27(–29)	(6.1–)8–11(–13)
Lateral leaflet length in upper cauline leaf	18.6±4.1	7.1±2.3
	(12.5–)14–25(–27)	(2.5–)5–10.2(–11)
Lateral leaflet width in upper cauline leaf	3.2±1.1	1.7±0.6
	(2–)2.2–4(–7)	(0.9–)1.1–2.5(–2.8)
Number of inflorescences for each stem	2–5(–10)	1–2
Bract shape and apex	narrowly triangular and acute to apiculate	more or less parallel-sided and obtuse
Bract length	18.4±3.9	8.8±1.8
	(12–)14–23(–26)	(5.8–)6.5–11(–12)
Bract lobe length	15.4±4.4	5.3±1.6
	(10–)11–21.5(–24)	(3.2–)3.9–7.5(–9)
Flower colour	light yellow or flushed with pink	purplish-pink or cream flushed with pink
Standard length	9.1±0.7	11.8±1.2
	(7.9–)8.5–9.9(–10.3)	(10–)10.4–13.6(–13.8)
Standard width	2.9±0.3	3.8±0.7
	(2.3–)2.6–3.3(–3.8)	(2.9–)3.2–5.1(–5.4)
Wing length	9.3±0.6	11.5±1.3
	(8.1–)8.8–10.1(–10.5)	(9.2–)10.1–13.1(–13.8)
Wing width	1.3±0.2	2.0±0.5
	(1–)1.2–1.6(–1.7)	(1.4–)1.5–2.6(–3.2)
Wing claw	6.3±0.4	7.3±0.8
	(5.3–)5.8–6.8(–7)	(6–)6.3–8.2(–9)
Keel length	9.6±0.7	11.5±1.2
	(8–)9–10.5(–10.8)	(9.7–)10.2–12.9(–13.7)
Staminal tube length (without free filaments and anthers)	8.0±0.5	9.5±1.0
	(6.6–)7.3–8.7(–8.8)	(8.2–)8.3–10.8(–11)
Ratio bract length/standard length	2.0±0.4	0.7±0.1
	(1.5–)1.6–2.3(–2.5)	0.6–0.8(–0.9)
Ratio bract length/calyx length	2.0±0.4	0.8±0.1
	(1.4–)1.6–2.3(–3.0)	(0.6–)0.7–1.0(1.1)

**Additional specimens examined. *Anthyllis apennina* (paratypes)**. **Italy. Lazio**: Campo di Grano, La Brecciosa (Pescosolido), pascolo, 1700 m a.s.l., 31/07/1995, *F. Conti* (APP No. 30831); La Brecciosa (Pescosolido), pascolo, 1700 m a.s.l., 31/07/1995, *F. Conti* (APP No. 30833); sentiero Forca di Fao, M. Arcione, M. Boragine (Cittareale, Rieti), pascoli secondari, 1619–1809 m a.s.l., 17/06/2016, *F. Conti*, *F. Bartolucci & R. Pennesi* (APP No. 57482); da Selvarotonda a Forca di Fao (Cittareale, Rieti), pascoli secondari, lembi di faggeta, 1533–1614 m a.s.l., 17/06/2016, *F. Conti*, *F. Bartolucci & R. Pennesi* (APP No. 57525); **Abruzzo**: M. Ocre presso l’Acquazzese, pascoli aridi, ca.1400 m a.s.l. 21 /07/2014, *F. Conti. B. Petriccione & G. Serafini s.n.* (APP Nos 55539, 55537, 55538, 55540, 55541, 55542, 55543, 55544); Prati del Sirente (Secinaro), pascoli aridi, 1150 m a.s.l., 07/06/2016, *L. Di Martino & F. Bartolucci* (APP Nos. 59702, 59703, 59704, 59705, 59706, 59707, 59708, 59709, 59710, 59711); M. Calvo, sia Lazio che Abruzzo, presso i ripetitori (Scoppito), pascoli aridi, 1460 m a.s.l., 04/06/2017, *F. Conti & V. Giacanelli* (APP Nos. 59729, 59730, 59801); V.ne di Sevice (Magliano de’ Marsi), 28/06/2017, *F. Conti* (APP No. 59802); al valico tra S. Colombo e Santo Stefano di Sessanio, lato destro (Barisciano), pascoli aridi, 1300 m a.s.l., 18/06/2017, *F. Conti* (APP Nos. 59872, 59873, 59874); Colle Biferno, PNALM (Ortona dei Marsi), pascoli aridi, 1300 m a.s.l., 13/06/2017, *F. Conti & F. Bartolucci* (APP Nos. 59887, 59888); Piani di Pezza fino al laghetto (Rocca di Mezzo), pascoli aridi, 1460 m a.s.l., 19/04/2019, *F. Conti*, *E. Proietti*, *I. Eckersley*, *C.Oberprieler & R. Vogt* (APP Nos. 64972, 64973, 64974); Prati del Sirente, vicino alla stazione di *Iris marsica* (Secinaro), radure di cerrata, 1110 m a.s.l., 19/06/2019, *F. Conti*, *E. Proietti*, *I. Eckersley*, *C. Oberprieler & R. Vogt* (APP Nos. 64979, 64980); versante meridionale di Pizzo Camarda (L’Aquila), pascoli aridi, 1500 m a.s.l., 16/07/2019, *F. Conti*, *F. Bartolucci & E. Proietti* (APP Nos. 65148, 65149, 65150).

***Anthyllis pulchella*. Italy. Lazio**: Campo di Grano, La Brecciosa (Pescosolido), pascolo, 1700 m, 31/07/1995, *F. Conti* (APP Nos. 30831, 30833); sentiero Forca di Fao, M. Arcione, M. Boragine (Cittareale), pascoli secondari, 1619–1809 m, 17/06/2016, *F. Conti*, *F. Bartolucci & R. Pennesi* (APP No. 57492); **Abruzzo**: M. Secine presso la vetta (Ateleta), pascoli, 1700–1800 m, 19/06/1997, *F. Conti* (APP No. 13485); Monte Argatone, Serra della Terratta, (Scanno), cresta, 2000–2200 m, 28/06/1995, *F. Conti* (APP No. 30832); M. Genzana (Scanno), prati alti, 2000 m, 19/07/1997, *F. Conti* (APP No. 35964); ibidem, 07/07/1997, *F. Conti* (APP No. 36077); Monte Greco – La Capriola (Barrea), 02/08/2001, *F. Conti* (APP No. 48654); V.ne di Sevice (Magliano De’ Marsi), 28/06/2017, *F. Conti* (APP No. 59800); Montagna dei Fiori (Valle Castellana), 05/06/2019, *F. Conti* (APP Nos. 64950, 64951); Piani di Pezza fino al laghetto (Rocca di Mezzo), pascoli aridi, 1460 m m, 19/04/2019, *F. Conti*, *E. Proietti*, *I. Eckersley*, *C.Oberprieler & R. Vogt* (APP No. 64970); **Montenegro.** Durmitor, Savin Kuk – Sljeme, substrato calcareo, 2000–2350 m, 08/07/1996, *D. Lakusic*, *F. Conti & G. Tomovic*, (APP No. 9082); Monte Prokletije, Festuco-Seslerietea, limestone, 1920 m, 16/07/2003, *D. Lakušić*, *F. Conti*, *Z. Bulić*, *M. Niketić*, *G. Ciaschetti*, *G. Tomović & S. Adžiablahović* (APP No. 31711); Montenegro, Lovcen, 6000, 5/06/1867, *Huter s.n.* (lectotype PAD barcode HD08734!; isolectotype B barcode B101113247, BM barcodes BM000751284 and BM000751285); Monte Orjen, 15/07/1868, *Th. Pichler* (PAD barcode HD08733); **Albania.** M. Çika tra Llogara e la vetta, pendii rupestri, 1020–1990 m, 23/06/2015, *F. Conti*, *D. Lakušić*, *R. Di Pietro*, *N. Kuzmanović*, *A. Stinca*, *S. Đurović*, *I. Janković & R. Pennesi* (APP Nos. 56418, 56477, 56498); tra Poliçan e M. Nemërçkë, pendii rupestri e pascoli, 1200–2480 m, 26/06/2015, *F. Conti*, *D. Lakušić*, *R. Di Pietro*, *N. Kuzmanović*, *A. Stinca*, *S. Đurović*, *I. Janković & R. Pennesi* (APP No. 56949, 57145); **Greece.** M. Olimpo, presso il Rifugio, 2200 m, 05/08/2009, *F. Conti & D. Uzunov* (APP No. 56699), *ibidem*, 2300–2700 m, 06/08/2009, *F. Conti & D. Uzunov* (APP No. 56727); **Turkey.** Uludag presso la vetta 2436 m, 09/07/2006, *F. Conti & D. Uzunov* (APP No. 56764).

**Anthyllis vulneraria subsp. nana. Italy**. **Marche**: Monte Vettore presso la cima 24/07/2013, *F. Conti*, *A. Manzi & P. Minghetti* (APP No. 62414); **Lazio**: sopra le prese dell’ENEL del T. Molinaro (Amatrice), Pascolo, 1350–1600 m, 12/06/2014, *F. Bartolucci & F. Conti* (APP No. 54814); F.so Piè di Lepre (Amatrice), pascoli, 1450–1800 m, 12/06/2014, *F. Bartolucci & F. Conti* (APP No. 54601); Terminillo a W di Sella Iacci 1700 m, 10/06/2003, *F. Conti & G. Gottschlich* (APP No. 63522); **Abruzzo**: Gruppo del Gran Sasso, Corno Grande presso la Sella del Brecciaio (L’Aquila), praterie altitudinali, 2400–2500 m, 21/07/1999, *F. Conti*, *D. Lakusic & Ph. Küpfer* (APP No. 1048); Lago di Campotosto (Campotosto), pascoli aridi, incolti, 1315–1350 m, 30/05/1999, *D. Tinti* (APP Nos. 2602–2604); Caselle, bivio Vado di Corno (L’Aquila), festuceti e brachipodieti, 1820 m, 13/06/2003, *F. Conti et al.* (APP No. 7153); Vallone di Selva Romana (Pennapiedimonte), pascoli – rupi, 1575 m, 22/06/2004, *F. Conti & F. Bartolucci* (APP Nos. 10726, 10727, 10736); Blockhaus-Monte Focalone (Pennapiedimonte-Caramanico Terme), pascoli sassosi, 2200 m, 28/07/2004, *G. D’Orazio* (APP No. 12216); loc. Il Pratuccio (Sant’Eufemia a Majella), pascoli sassosi, 1400 m, 22/07/1987, *F. Conti & G. Pirone* (APP No. 13309); Monte Ocre, vallone Canavine, dal bordo superiore della faggeta alla conca di Settacque (Rocca di Cambio), pascoli sassosi, 1570 m, 22/06/2005, *F. Conti*, *F. Bartolucci*, *L. Bernardo*, *D. Iamonico*, *M. Latini*, *R. Lorenzetti*, *I. Londrillo*, *E. Pellegrini*, *N. Ranalli*, *L. Peruzzi*, *E. Scassellati*, *D. Tinti & V. Viscosi* (APP No. 15317); M. Sirente, tra la faggeta e Val Lupara (Secinaro), pendii rupestri, 1970 m, 20/06/2005, *F. Conti*, *F. Bartolucci*, *L. Bernardo*, *D. Di Santo*, *D. Iamonico*, *M. Latini*, *R. Lorenzetti*, *N. Ranalli*, *L. Peruzzi*, *D. Tinti & V. Viscosi* (APP No. 15497); Monte Sirente, valle Majori (Secinaro), Pendii rupestri, 1500–2100 m, 20/06/2005, *F. Conti*, *F. Bartolucci*, *L. Bernardo*, *D. Di Santo*, *D. Iamonico*, *M. Latini*, *R. Lorenzetti*, *N. Ranalli*, *L. Peruzzi*, *D. Tinti & V. Viscosi* (APP No. 15549); Gran Sasso, tra Vado di Corno e Valle dell’Inferno (Isola del Gran Sasso), Pascoli, 1950 m, 17/07/2004, *F. Conti & F. Bartolucci* (APP No. 16617); Gran Sasso – M. Camicia, versante occidentale (Castel del Monte), pascoli, 1800–2000 m, 21/07/1997, *F. Conti* (APP No. 17536); Gruppo della Laga – Valle del Rio Castellano, Vado di Annibale – Cima Lepri (Valle Castellana), pascoli e praterie, 2119 m, 22/06/2005, *S. Cecchetti* (APP No. 21299); Gruppo della Laga – Valle del Rio Castellano, Macera della Morte – Pizzitello (Rocca S. Maria), pascoli e praterie, 2200 m, 19/07/2005, *S. Cecchetti* (APP No. 21300); Gruppo della Laga – Valle del Rio Castellano, Pianaccio (Valle Castellana), praterie, pascoli sassosi, alvei di torrente, 1850–2090 m, 14/07/2005, *F. Conti & S. Cecchetti* (APP No. 21301); Gruppo della Laga – Valle del Rio Castellano, cascata del Diedro – Pedatelle (Rocca S. Maria), rupi arenacee, pascoli, praterie, 1730 m, 23/06/2005, *S. Cecchetti* (APP No. 21302); M. di Valle Caprara, Costa dell’Ortella, Gioia dei Marsi (Lecce nei Marsi), pascoli, 1800–1900 m, 15/06/1997, *F. Conti & F. Minutillo* (APP No. 33406); Monte Focalone (Fara San Martino), 2100–2500 m, 08/08/2008, *F. Conti*, *J. Meister & F. Minutillo* (APP No. 36451); Serra Lunga (Villavallelonga), pascoli e faggeta, 1759 m, 15/06/2010, *F. Bartolucci & N. Ranalli* (APP No. 50080); Campo Pericoli (Pietracamela), Pascoli sassosi, 2200 m, 23/07/2009, *F. Bartolucci & M. Iocchi* (APP No. 50417); Pizzo Cefalone (L’Aquila), Pascolo – rupi, 2200–2400 m, 20/06/2013, *A. Stinca & F. Bartolucci* (APP No. 52707); Monte Camicia, south east flank above Rif. Fonte Vetica (Castel del Monte), pascoli, rupi, 1600–2500 m, 16/07/2013, *A. Stinca & F. Bartolucci* (APP No. 52759); tra la Sella di M. Aquila e la cresta del Duca (L’Aquila), pascoli – rupi, 2200–2300 m, 25/06/2013, *A. Stinca & F. Bartolucci* (APP No. 52774); presso le Gondole (Santo Stefano di Sessanio), pascoli aridi, 10/05/2013, *F. Conti* (APP No. 52955); da Campo Imperatore a M. Aquila (L’Aquila), pascoli e rupi, 02/07/2013, *A. Stinca & F. Bartolucci* (APP No. 53141); Brancastello (L’Aquila), Pascoli, 22/08/2014, *F. Conti* (APP Nos. 54508, 54511, 54512, 54515); Vallone Fossaceca (Isola del Gran Sasso d’Italia), pascolo, 1200–2200 m, 31/07/2014, *F. Conti & F. Bartolucci* (APP Nos. 54837, 54838); **Molise**: vetta del Monte a Mare (Pizzone), ghiaione, 2160 m, 14/06/1993, *F. Conti* (APP No. 30837); Alta Valle Pagana (Pizzone), pascolo sommitale, 1900 m, 14/06/1993, *F. Conti* (APP No. 30838); Valle Fredda, sopra il limite del bosco (Pizzone), prato, 1800–2000 m, 06/07/1993, *F. Conti* (APP No. 30839); La Metuccia, Monte a Mare (Pizzone), pascolo, 1900 m, 08/07/1992, *F. Conti* (APP No. 30840); Valle Fredda (Pizzone), pascolo sommitale, 1800–2000 m, 16/06/1993, *F. Conti* (APP No. 30841); Valle Pagana ed altro circo glaciale adiacente (Pizzone), pascolo, 1900 m, 23/07/1991, *F. Conti* (APP No. 30842).

**Anthyllis vulneraria subsp. vulnerarioides. Spain.** de Castanezé à Malibierun, 10/08/1893, *Saubadre* (P barcode P03056343); **France.** Monêtier-les-Bains à Guy Chevalier, pelouses rocailleuses, 2000 m, 21/08/1901, *A. Faure* (GAP barcode GAP046961); *ibidem*, 2200 m, 25/07/1905, *A. Faure* (P barcode P03622952); Monêtier-les-Bains, paturages vers 2200 m, 21/08/1901, *A. Faure* (P barcode P03069103); *ibidem*, 5/08/1896 (P barcode P03069104); **France/Italy**: Mont Cenis, s.d., s.coll. (GAP barcode GAP042327); Mont Cenis à Savalin, *Huguenin 498* (GAP barcode GAP037228); Moncenis (patta creusa), 1838, *s.coll.* (TO, epitype); Bardonecchia, Valfroide […] de Aigle, 30/07/1899, *Ferrari* (TO); Mt. Cenis, 1838, *Bonnar* (TO).

## Supplementary Material

XML Treatment for Anthyllis apennina
